# Exploring the mechanism of Shengmai Yin for coronary heart disease based on systematic pharmacology and chemoinformatics

**DOI:** 10.1042/BSR20200286

**Published:** 2020-06-10

**Authors:** Yan Jiang, Qi He, Tianqing Zhang, Wang Xiang, Zhiyong Long, Shiwei Wu

**Affiliations:** 1 Department of General Surgery, The First Affiliated Hospital of University of South China, Hengyang, Hunan Province, China; 2 Graduate College, Hunan Normal University, Changsha, Hunan Province, China; 3 Intensive Care Unit, People’s Hospital of Ningxiang City, Ningxiang 410600, Hunan Province, China; 4 Graduate College, University of South China, Hengyang, Hunan Province, China; 5 Department of Cardiology, The First Affiliated Hospital of University of South China, Hengyang, Hunan Province, China; 6 Graduate College, Guilin Medical University, Guilin, Guangxi Province, China; 7 Department of Rheumatology, Affiliated Hospital of Guilin Medical University, Guilin, Guangxi Province, China; 8 Department of Physical Medicine and Rehabilitation, Guangdong General Hospital, Shantou University Medical College, Shantou, Guangdong, China; 9 Graduate College, Shantou University Medical College, Shantou, Guangdong Province, China; 10 Department of Traditional Chinese Medicine, The Eighth Affiliated Hospital, Sun Yat-sen University, Shenzhen, Guangdong Province, China

**Keywords:** Chemoinformatics, Chinese medicine, Coronary heart disease, Shengmai Injection, Shengmai Yin, Systematic pharmacology

## Abstract

**Objective:** To explore the mechanism of Shengmai Yin (SMY) for coronary heart disease (CHD) by systemic pharmacology and chemoinformatics.

**Methods:** Traditional Chinese Medicine Systems Pharmacology Database (TCMSP), traditional Chinese medicine integrative database (TCMID) and the traditional Chinese medicine (TCM) Database@Taiwan were used to screen and predict the bioactive components of SMY. Pharmmapper were utilized to predict the potential targets of SMY, the TCMSP was utilized to obtain the known targets of SMY. The Genecards and OMIM database were utilized to collect CHD genes. Cytoscape was then used for network construction and analysis, and DAVID was used for Gene Ontology (GO) and pathway enrichment analysis. After that, animal experiments were then performed to further validate the results of systemic pharmacology and chemoinformatics.

**Results:** Three major networks were constructed: (1) CHD genes’ protein–protein interaction (PPI) network; (2) SMY–CHD PPI network; (3) SMY known target–CHD PPI network. The other networks are minor networks generated by analyzing the three major networks. Experimental results showed that compared with the model group, the Shengmai injection (SMI) can reduce the myocardial injury score and the activities of serum aspartate aminoconvertase (AST), CK and lactate dehydrogenase (LDH) in rats (*P*<0.05), and reduce serum lipid peroxide (LPO) content and increase serum superoxide dismutase (SOD) and glutathione peroxidase (GSH-Px) activities in myocardial infarction rats (*P*<0.05). SMI can also decrease the expression of MMP-9 mRNA and increase that of TIMP-1 mRNA (*P*<0.01).

**Conclusion:** SMY may regulate the signaling pathways (such as PPAR, FoxO, VEGF signaling), biological processes (such as angiogenesis, blood pressure formation, inflammatory response) and targets (such as AKT1, EGFR, MAPK1) so as to play a therapeutic role in CHD.

## Introduction

Coronary heart disease (CHD) is a heart disease caused by coronary atherosclerosis [1,2]. Epidemiological surveys show that 7.3 million people worldwide currently die of ischemic heart disease each year, ranking first among all diseases, accounting for 12.8% of all diseases [3]. Especially in China, patients with CHD increase at an annual rate of 20%, and the number of deaths is approximately 10–20% of all heart diseases [4]. At present, the Western medicine treatment of CHD is mainly based on comprehensive management. According to the patient’s disease stage, the treatment is mainly based on anti-platelet, anti-atherosclerosis and lipid-lowering stable plaques [5,6]. At present, in alternative medicine, traditional Chinese medicine (TCM) has gradually shown its unique advantages in the treatment of CHD. With the increase in the number of large-scale randomized controlled trials, the Chinese medicine formula has gradually become the first-line treatment measure for CHD treatment [7–9]. For example, Tongxinluo Capsule and Fufang Danshen Diwan can achieve a certain effect in the treatment of CHD [8,9].

Shengmai Yin (SMY) is derived from Yixue Qiyuan written by Zhang Yuan of the Jin Dynasty, and is one of the classic prescriptions of traditional Chinese medicine. SMY is composed of Renshen (*Panax ginseng, Araliaceae*), Maidong (*Ophiopogon japonicas, Liliaceae*) and Wuweizi (*Schisandra chinensis, Schisandraceae*), which has the effects of regulating qi and phlegm, biliary and stomach, and can be used to treat heart palsy and chest pain caused by phlegm and blood stasis obstruction. Evidence-based medicine shows that the use of SMY can effectively prevent and treat CHD [10–12]. Modern pharmacological studies have shown that SMY can improve myocardial blood supply, heart failure and myocardial fibrosis, thereby reducing the symptoms of CHD [13–16]. However, due to the complexity of the Chinese medicine formula and the comprehensive nature of the occurrence and development of CHD, SMY’s mechanism has not yet been comprehensively explained.

Systematic pharmacology can analyze the mechanism of Chinese medicine formula in regulating signaling pathways from the perspective of system biology and biological network balance, based on chemical informatics, computer virtual computing and database retrieval. This methodology can clarify the ‘Compound-target-pathway’ network of Chinese medicine formula-disease, reveal the mechanism of Chinese medicine formula for treating diseases, improve the therapeutic effect of drugs, reduce toxic and side effects, and save the cost of drug development [17–21]. Meanwhile, systemic pharmacology is in line with the concept of TCM’s systemic treatment, and fits the path of TCM toward modernization [17–20]. Therefore, we hope to analyze the mechanism of SMY treatment of CHD through systematic pharmacological methods, build a ‘compound-target-pathway’ network, and provide research ideas and theoretical basis for further studying the mechanism of SMY treatment of CHD.

## Materials and methods

### SMY bioactive compounds prediction

The keywords *Panax ginseng, Araliaceae, Ophiopogon japonicas, Liliaceae* and *Schisandra chinensis, Schisandraceae* were utilized in the traditional Chinese medicine integrative database (TCMID, http://www.megabionet.org/tcmid/) [22], the traditional Chinese Medicine Database@Taiwan (http://tcm.cmu.edu.tw/zh-tw/) [23] and the Traditional Chinese Medicine Systems Pharmacology Database (TCMSP™, http://tcmspw.com/tcmsp.php) [24] to search the compound of each herb. The ADME model was used to predict the bioactive components in each herb, with the standard: oral bioavailability (OB) ≥ 30%, Caco-2 permeability > −0.4 and drug-likeness (DL) ≥ 0.18 [17–20,25–28]. Finally, combined with our previous HPLC-Q-TOF-MS experiments and other reference [29–33], a total of 40 compounds were obtained.

### Potential targets prediction and known targets collection

The structures of potential compounds were constructed by ChembioOffice 14.0 according to the structural formula found in the SciFinder (http://scifinder.cas.org), and saved as ‘mol2’ files. The ‘mol2’ files were imported into Pharmmapper (http://lilab-ecust.cn/pharmmapper/) to predict the potential targets of each compound [34,35]. This database uses a reverse pharmacophore matching method to obtain a drug database target with an active small molecule probe so as to predict its biological activity [34,35]. The known targets were collected from the TCMSP [24].

The protein name of the target was entered into the UniProt database (http://www.uniprot.org/) and limit the search conditions to ‘Reviewed (Swiss-Prot)’ and ‘*Homo sapiens*’ so as to standardize the results. Eventually, the official symbol of each target was obtained. The details are described in Supplementary Tables S1 and S2.

### CHD genes

The CHD-related genes were obtained from CHD@ZJU Database ver. 3.0 (http://tcm.zju.edu.cn/chd/) [36], Online Mendelian Inheritance in Man (http://omim.org/) [37] and Genecards (http://www.genecards.org) [38]. The keywords were ‘Coronary Diseases’, ‘Disease, Coronary’, ‘Diseases, Coronary’, ‘Coronary Heart Disease’, ‘Coronary Heart Diseases’, ‘Disease, Coronary Heart’, ‘Diseases, Coronary Heart’, ‘Heart Disease, Coronary’, ‘Heart Diseases, Coronary’ (see Supplementary Table S3).

### Protein–protein interaction data

The String database (http://string-db.org/) were utilized to get the data of protein–protein interaction (PPI). While searching the String database, the species limited to ‘*Homo sapiens*’ with confident score ≥ 0.4 [17–20,39]. The node interaction type is default.

### Network construction and analysis method

The potential compounds, potential targets, CHD genes and PPI data were imported into Cytoscape 3.7.0 software (http://cytoscape.org/) for network construction [40]. In the network, a node represents a compound, a target, a pathway or so on [40]. Nodes are connected by edges; depending on the network being constructed, the meaning of edges is different [40]. Degree represents the number of connections (edges) of a node in the network [40].

Three major networks and several minor networks were constructed. The three major networks were: (1) CHD genes’ PPI network; (2) SMY–CHD PPI network; (3) SMY known target–CHD PPI network. The other networks are minor networks generated by analyzing the three major networks.

Meanwhile, the networks were analyzed by the plugin MCODE to obtain cluster [41,42]. Additionally, the DAVID ver 6.8 (https://david-d.ncifcrf.gov) was utilized to undergo Gene Ontology (GO) enrichment analysis and pathway enrichment analysis [43].

### Experimental materials

#### Experimental animal

SD rats, weighing 230–250 g, half male and half female, were purchased from Changsha Tianqin Biotechnology Co., Ltd., license number: SCXK (Xiang) 2014-0011. The experimental animals were housed in a light and dark cycle at 22°C for 12 h, and allowed to eat and drink freely. All animal experimental procedures were strictly performed in accordance with the guidelines of the Animal Ethics Committee of Hunan Normal University and the guidelines for the care and use of experimental animals (approval number: 2016021).

#### Drugs and reagents

Shengmai injection (SMI) (10 ml/branch, ginsenoside Rg1 concentration should not be less than 0.08 mg/ml, ginsenoside concentration Re should not be less than 0.04 mg/ml), was provided by Jiangsu Suzhong Pharmaceutical Group Co., Ltd., batch number 20161104, and was formulated with 5% glucose injection to the required concentration. Glutathione peroxidase (GSH-Px) assay kit (colorimetric method) was provided by Shanghai Junrui Biotechnology Co., Ltd. (UFJC0212). Superoxide dismutase (SOD) detection kit was provided by Beijing Biodi Biotechnology Co., Ltd. (ESOD012). The lipid peroxide (LPO) kit was provided by Nanjing Jiancheng Biotechnology Research Institute (A107). TRIzol Reagent (provided by Invitrogen Life Technologies, lot number: 15596-026); real-time PCR instrument (Shanghai Hongshi Medical Technology Co., Ltd.); PCR kit, PCR primers (provided by Wuhan Google Biological Reagent Co., Ltd.) (Table 1).

**Table 1:** Primer design

Gene primer sequence	Product	Size (bp)
*MMP-9*	F: GCAAACCCTGCGTATTTCCATT	283
	R: GCGATAACCATCCGAGCGAC	
*TIMP-1*	F: TAAAGCCTGTAGCTGTGCCC	326
	R: CATAACGCTGGTATAAGGTGGTC	
*GAPDH*	F: TTCCTACCCCCAATGTATCCG	482
	R: CATGAGGTCCACCACCCTGTT	

### Experimental methods

#### Animal modeling, grouping and intervention

Sixty rats were randomly divided into a sham operation group (Sham), CHD model group (model), SMI low-dose group (SMIL), and SMI high-dose group (SMIH), with 12 in each group. According to the clinical daily dose of SMI (30 ml/60 kg), the dosage of rats was converted according to the equivalent dose conversion table of human and rat body surface area. The SMIL was injected SMI 5.0 ml/kg intraperitoneally, the SMIH was injected SMI 10.0 ml/kg intraperitoneally, and the sham operation group and the CHD model group were given 5% glucose injection 10.0 ml/kg.

Coronary artery ligation in rats is performed according to the method provided in [44,45]: under ether anesthesia, the rat was fixed on the operating table in the supine position, thoracotomy was opened from the left 3–4 intercostal space, the heart was exposed, and the left anterior descending coronary artery was found between the pulmonary artery cone and the left atrium. The sham operation group was only threaded and not ligated. The rest of the groups were immediately ligated to the coronary artery with line 0, the heart was returned to the chest, and the blood and gas in the chest were squeezed out, and finally the chest was closed quickly. The chest opening time should not exceed 30 s.

Animals in each group were administered once by intraperitoneal injection immediately after surgery, and once again after 12 h. After the coronary artery was ligated for 24 h, the rats were anesthetized by intraperitoneal injection with 30 mg/kg sodium pentobarbital, and blood was taken from the abdominal aorta. During the experiment, all animals that died due to surgery, anesthesia and other reasons, and that the survival time after the operation did not meet the requirements of the material collection time, were not included in the experimental observation items.

#### Histopathology of myocardium in rats

After the rats were killed by cervical dislocation, the tissues of the myocardial infarction area were taken and immersed in a 4% paraformaldehyde solution for 24 h for fixation. The tissues were then removed and routinely dehydrated, clear, wax-embedded, and sectioned for Hematoxylin–Eosin (HE) staining. Observe and analyze the pathological changes of myocardium in rats, and score the degree of myocardial tissue damage according to myocardial morphology changes under light microscope: (1) if the myocardial fibers are neatly arranged and there is no necrosis and no inflammatory cell infiltration, it is recorded as ‘0’ point; (2) if the myocardial fibers have coagulative necrosis with mild infiltration of inflammatory cells, it is recorded as ‘1’ point; (3) if the myocardial fibers have a large number of coagulative necrosis and there is severe infiltration of inflammatory cells, it is recorded as ‘3’ points. A computer image analysis system was used to determine the area of the infarcted area for each HE-stained slice, and finally the percentage of the myocardial infarction area to the cross-sectional area of the myocardium was calculated.

#### Detection of serum myocardial enzymes

Serum aspartate aminoconvertase (AST), phosphocreatine kinase (CPK) and lactate dehydrogenase (LDH) activities were detected by COBAS-FARA automatic biochemical analyzer.

#### Detection of serum oxidative stress metabolites

Serum LPO content, SOD, GSH-Px activity were measured with the corresponding kits.

#### Detection of myocardial MMP-9 mRNA and TIMP-1 mRNA expression by reverse transcription PCR

Take 100 mg of myocardial tissue and add 1 ml of TRIzol reagent, and extract total myocardial RNA according to the product instructions. Reverse transcription PCR (RT-PCR) system: 10× PCR Buffer 2.5 μl, 25 mol/l MgCl 21.5 μl, 10 mmol/l dNTP1 μl, Taq enzyme 0.2 U, cDNA 2 μl, primer amount 10 pmol (the total system is 25 μl). Reaction conditions: 94°C for 5 min, then enter the PCR cycle, pre-denatured at 94°C for 30 s, annealed at 60°C and 58°C for 30 s, extended at 72°C for 45 s, and cycle 33 times and extend for 5 min. Take 5 μl of the PCR product and run it on a 1.5% agarose gel, then scan it on a gel imaging system to determine the absorbance of each target gene and GAPDH amplification product, and calculate their ratios.

### Statistical analysis

The SPSS 19.0 statistical software system was used, and the measured data were expressed as mean ± standard deviation (x ± s), and comparison between groups was performed by analysis of variance (one-way ANOVA). *P*<0.05 was significant difference.

## Results

### CHD genes’ PPI network analysis

#### CHD genes’ PPI network

**Figure 1:**
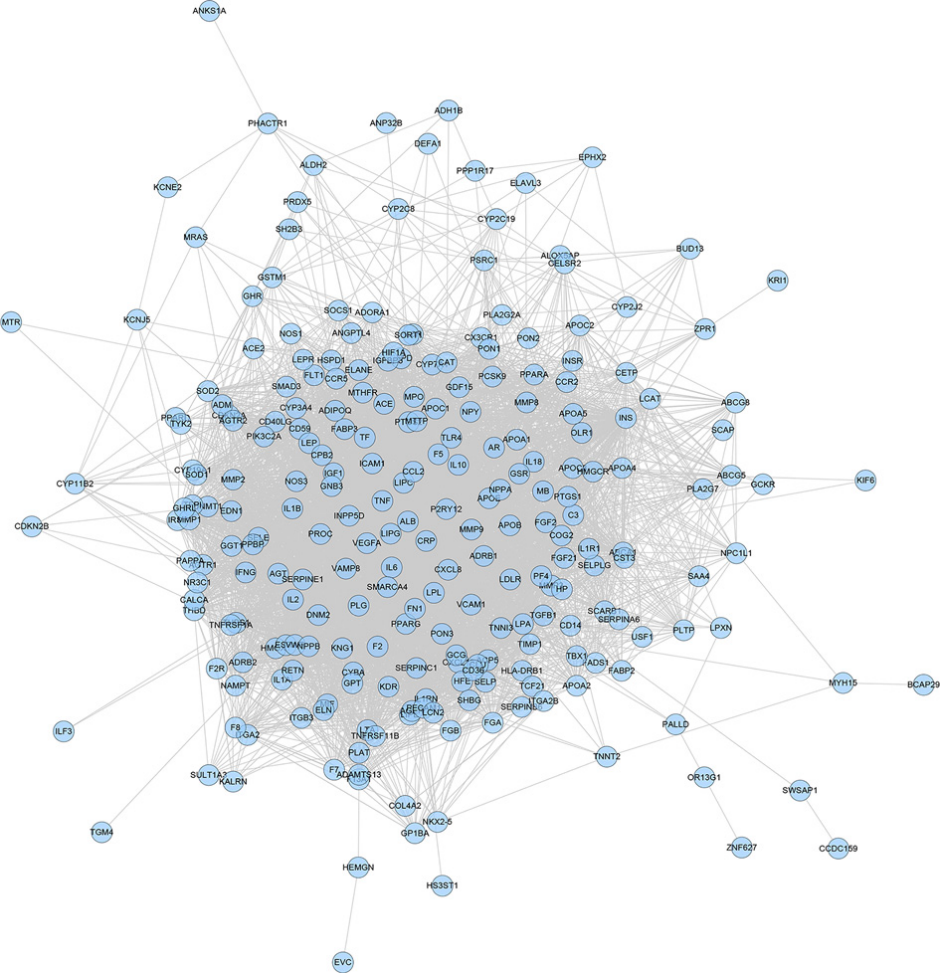
CHD genes’ PPI network

**Figure 2:**
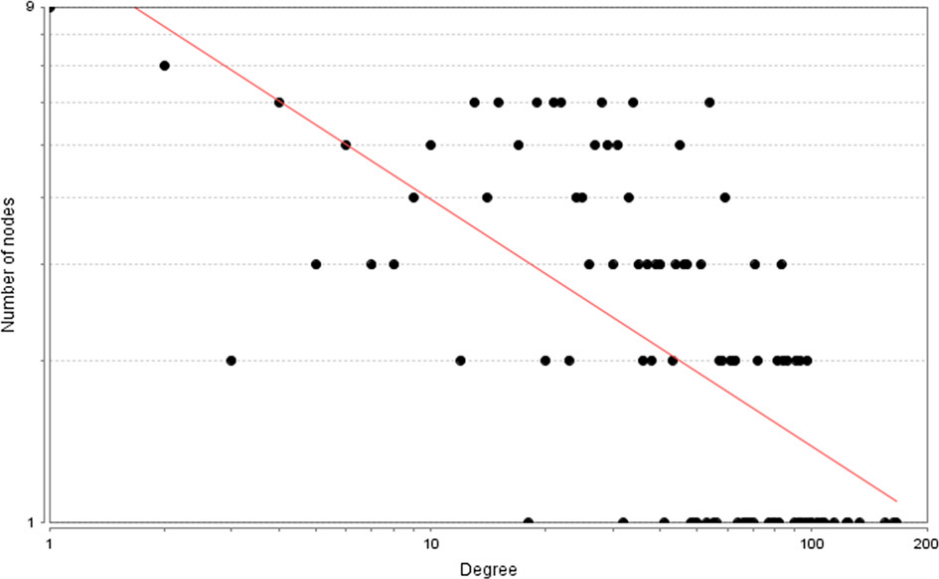
Node degree distribution of CHD genes’ PPI network

A total of 2683 CHD-related genes were obtained from the database. The genes with relevance score ≥ 1.5 were put into String database for PPI data. Finally, the CHD genes’ PPI network was constructed according to these information. This network consists of 247 CHD gene nodes and 4812 edges (Figure 1). These targets are arranged according to the degree from large to small. The top ten are: INS (167 edges), ALB (164 edges), interleukin-6 (IL-6) (156 edges), TNF (133 edges), C-reactive protein (CRP, 124 edges), VEGFA (123 edges), APOE (114 edges), CCL2 (108 edges), CXCL8 (106 edges), FN1 (103 edges). The topological property of this network was assessed by network analyzer tool, and the result demonstrates that CHD genes’ PPI network meets the power-law distribution (R^2^ = 0.445, y = 11.372x^−0.457^) (Figure 2), indicating that CHD genes’ PPI network is scale-free and has the general characteristic of biological network.

#### Clusters of CHD genes’ PPI network

**Figure 3:**
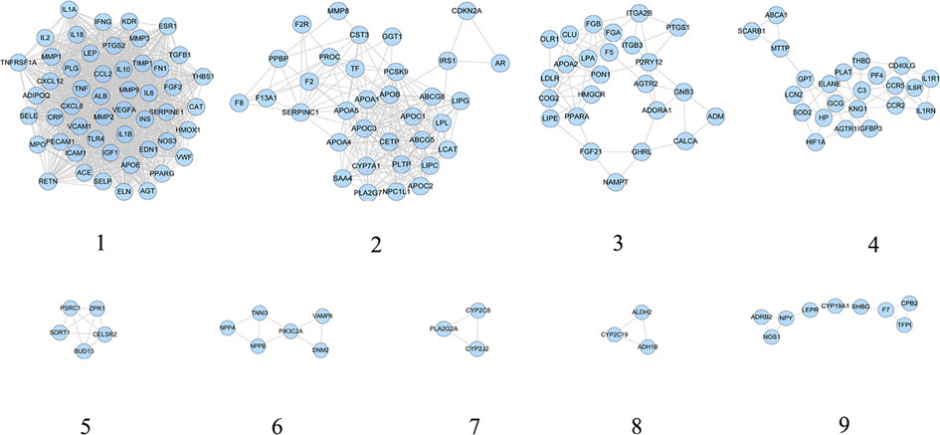
Cluster of CHD genes’ PPI network

CHD genes’ PPI network was analyzed by cytoscape’s plug-in MCODE and nine clusters were generated (Table 2 and Figure 3). The genes in clusters were put into DAVID for GO enrichment analysis.

**Table 2:** Clusters of CHD genes’ PPI network

Cluster	Score	Nodes	Edges	Genes
1	44.28	51	1107	ELN, VEGFA, THBS1, IL1B, PPARG, LEP, PECAM1, AGT, ICAM1, KDR, TIMP1, ESR1, MMP9, IL10, MMP2, IL18, CXCL12, CCL2, IGF1, TGFB1, FGF2, CAT, VCAM1, CXCL8, MMP1, IL2, APOE, HMOX1, IFNG, CRP, NOS3, PTGS2, ADIPOQ, IL1A, ACE, SERPINE1, SELP, INS, IL6, EDN1, TLR4, VWF, SELE, RETN, TNF, ALB, MPO, TNFRSF1A, MMP3, FN1, PLG
2	13.394	34	221	APOA1, PLTP, APOB, F2R, LPL, APOC3, ABCG8, F8, CETP, MMP8, F2, LIPC, CYP7A1, APOA5, APOC1, SAA4, LIPG, CDKN2A, PLA2G7, PROC, LCAT, CST3, APOA4, TF, IRS1, SERPINC1, PPBP, AR, NPC1L1, ABCG5, APOC2, PCSK9, F13A1, GGT1
3	5.917	25	71	GNB3, ADORA1, CALCA, LDLR, PPARA, PON1, ITGA2B, COG2, APOA2, P2RY12, PTGS1, LPA, HMGCR, LIPE, ITGB3, F5, FGF21, GHRL, AGTR2, OLR1, NAMPT, FGA, CLU, FGB, ADM
4	5.091	23	56	GCG, IL6R, CCR2, SOD2, ABCA1, LCN2, HP, C3, MTTP, IL1RN, ELANE, THBD, CCR5, HIF1A, PF4, CD40LG, SCARB1, IGFBP3, IL1R1, KNG1, AGTR1, PLAT, GPT
5	4.5	5	9	PSRC1, BUD13, ZPR1, SORT1, CELSR2
6	3.2	6	8	NPPA, TNNI3, PIK3C2A, NPPB, DNM2, VAMP8
7	3	3	3	CYP2C8, PLA2G2A, CYP2J2
8	3	3	3	CYP2C19, ALDH2, ADH1B
9	2.5	9	10	LEPR, ADRB2, CPB2, NPY, CYP19A1, F7, NOS1, TFPI, SHBG

After GO enrichment analysis, several biological processes were returned. Cluster 1 is associated with vasodilation, inflammatory response, coagulation, smooth muscle cell proliferation, hypoxia, and angiogenesis. Cluster 2 is involved in lipid metabolism. Cluster 3 and 9 are related to coagulation. Cluster 4 is involved in calcium regulation, inflammatory response, and foam cell formation. Cluster 7 is associated with redox. Cluster 5 did not return any biological processes. Cluster 6 and 8 failed to return any CHD-related biological processes. The details were described in Supplementary Table S4.

#### Pathway of CHD genes’ PPI network

**Figure 4:**
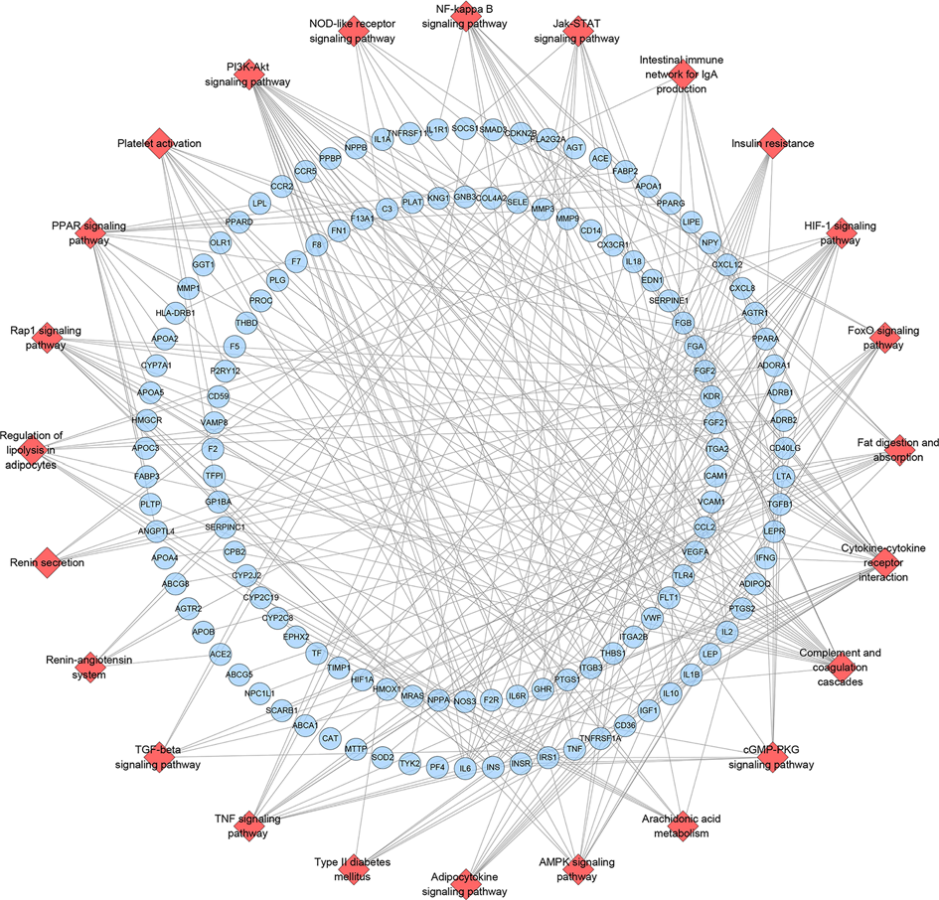
Signaling pathways of CHD Genes’ PPI Network (red diamond stands for signaling pathway; blue circle stands for CHD genes)

All CHD-related genes were put into DAVID to undergo pathway enrichment and 24 CHD-related signaling pathways were returned. These signaling pathways are arranged according to the degree of enrichment (negative correlation with *P*-value) and count from large to small. The top ten signaling pathways are: Complement and coagulation cascades (*P*=1.11*10^−14^, Count = 20), PPAR signaling pathway (*P*=1.70*10−^11^, Count = 17), Fat digestion and absorption (*P*=4.08*10−^09^, Count = 12), HIF-1 signaling pathway (*P*=5.00*10−^09^, Count = 17), Cytokine–cytokine receptor interaction (*P*=7.75*10^-09^, Count = 26), NF-κ B signaling pathway (*P*=3.61*10^−06^, Count = 13), Regulation of lipolysis in adipocytes (*P*=1.75*10^-05^, Count = 10), TNF signaling pathway (*P*=3.13*10^−05^, Count = 13), PI3K-Akt signaling pathway (*P*=5.90*10−^05^, Count = 24), Adipocytokine signaling pathway (*P*=5.94*10^-04^, Count = 9) (Figure 4) (see Supplementary Table S5).

### Potential compounds, potential targets and known targets

**Figure 5:**
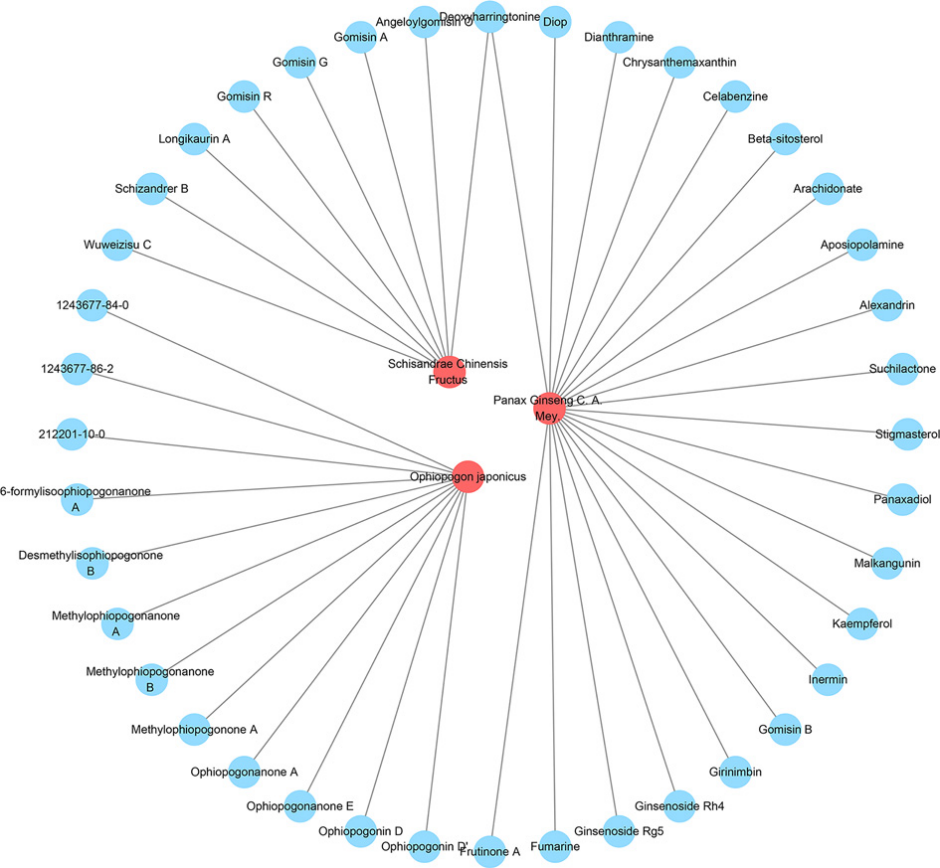
Herb-potential compounds network (red circle stands for herb; blue circle stands for potential compound)

Combined with our previous HPLC-Q-TOF-MS experiments and other references [29–33], a total of 40 potential compounds were obtained: ginsenoside Rh4, chrysanthemaxanthin, gomisin B, methylophiopogonanone B, panaxadiol, schizandrer B, 6-formylisoophiopogonanone A, celabenzine, diop, kaempferol, malkangunin, ophiopogonanone A, stigmasterol, β-sitosterol, fumarine, longikaurin A, methylophiopogonone A, wuweizisu C, angeloylgomisin O, arachidonate, ginsenoside Rg5, girinimbin, ophiopogonanone E, 5,7,4-trihydroxy-6-methyl homoisoflavanone (1243677-86-2), 5,7-dihydroxy-4-methoxy-6-methyl homoisoflavanone (212201-10-0), dianthramine, gomisin A, suchilactone, aposiopolamine, gomisin R, inermin, gomisin G, ophiopogonin D, 5,7,2,4-tetrahydroxy-8-methoxy-6-methyl homoisoflavanone (1243677-84-0), alexandrin, deoxyharringtonine, desmethylisophiopogonone B, frutinone A, methylophiopogonanone A, ophiopogonin D′. *Panax Ginseng C. A. Mey.* contains 21 compounds; *Schisandrae Chinensis Fructus* contains 8 compounds; *Ophiopogon japonicus* contains 12 compounds (Figure 5).

**Figure 6:**
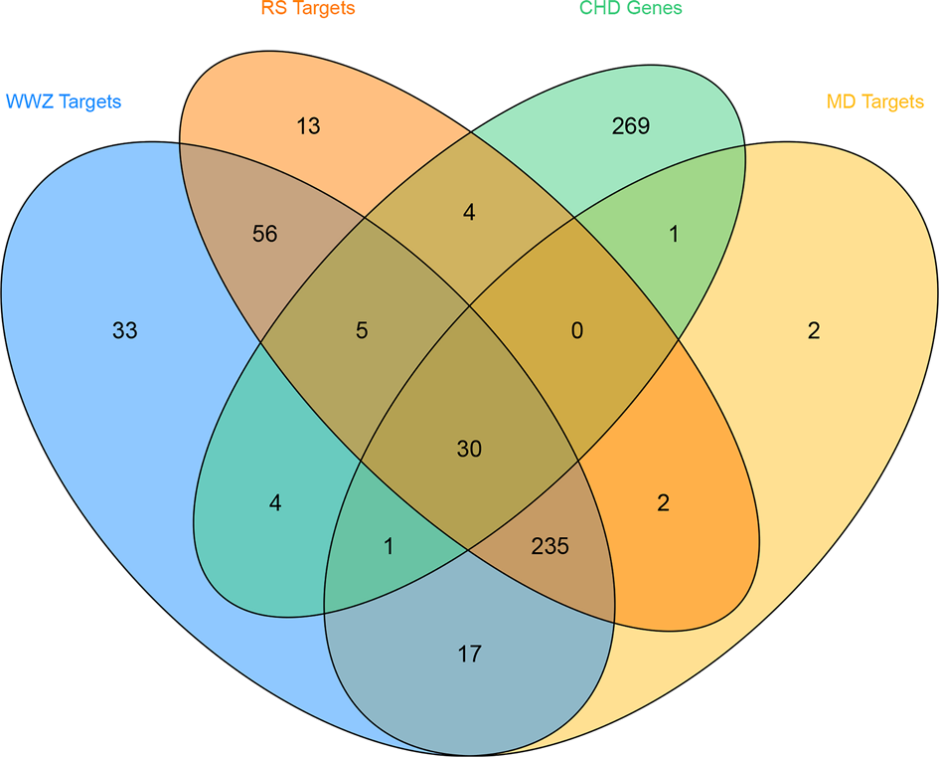
Venn diagram of potential targets (WWZ: *Schisandrae Chinensis Fructus*; RS: *Panax Ginseng C. A. Mey.*; MD: *Ophiopogon japonicus*.)

After the potential target prediction, a total of 400 potential targets were returned. *Panax Ginseng C. A. Mey.* contains 345 potential targets; *Schisandrae Chinensis Fructus* contains 381 potential targets; *Ophiopogon japonicus* contains 288 potential targets (Figure 6). Meanwhile, a total of 102 known targets were obtained.

### SMY–CHD PPI network analysis

#### SMY–CHD PPI network

**Figure 7:**
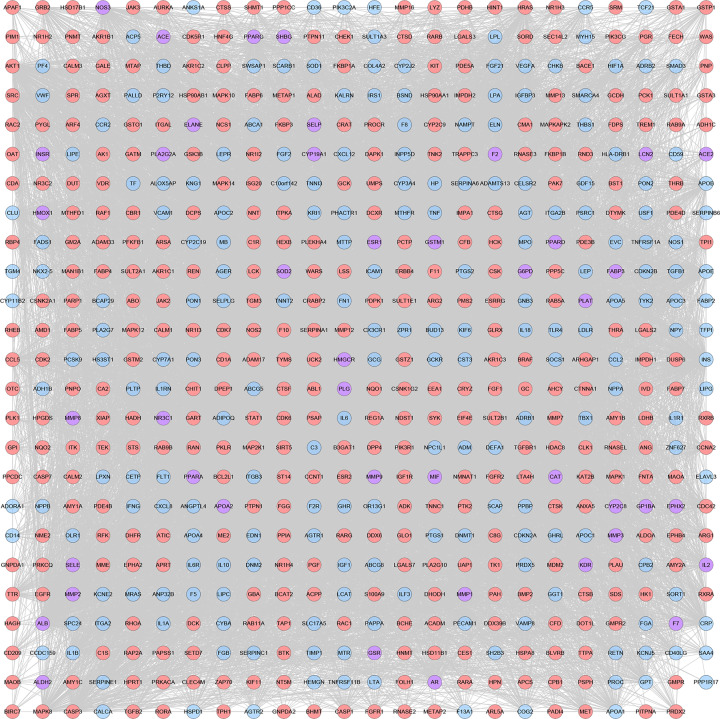
SMY–CHD PPI network (pink circle stands for SMY targets; blue circle stands for CHD targets; purple target stands for SMY-CHD targets)

**Figure 8:**
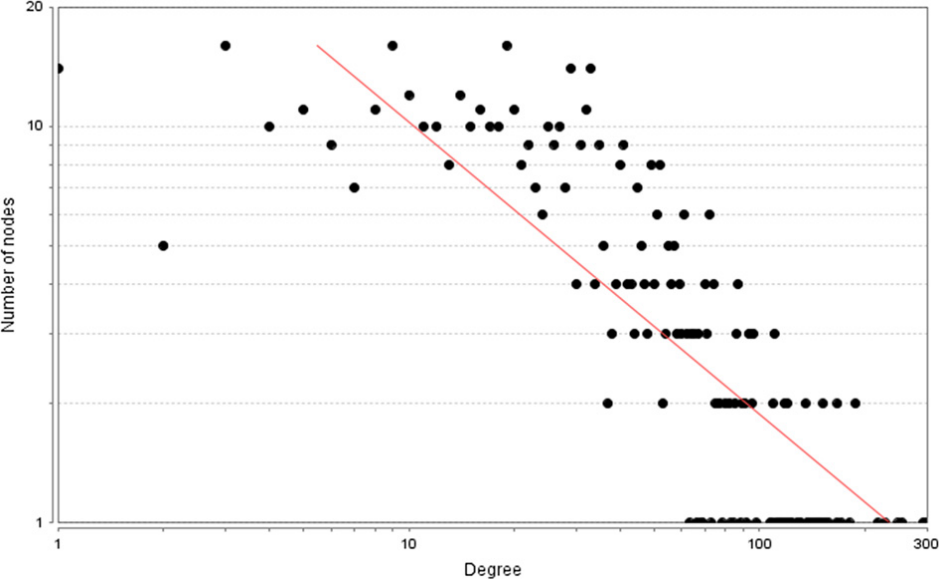
Node degree distribution of SMY–CHD PPI network

The potential targets and CHD genes were import into the String database to collect PPI data so as to build the SMY–CHD PPI network. This network contains 347 SMY target nodes, 209 CHD gene nodes, 43 SMY–CHD target nodes, 12755 edges (Figure 7). These targets are arranged according to the degree from large to small. The top 20 targets can be divided into three categories: (1) SMY targets: AKT1 (249 edges), EGFR (188 edges), MAPK1 (180 edges), SRC (166 edges), MAPK8 (154 edges), CASP3 (151 edges); (2) CHD genes: INS (298 edges), IL6 (255 edges), TNF (228 edges), VEGFA (217 edges), FN1 (188 edges), IGF1 (171 edges), CXCL8 (167 edges), CRP (157 edges), CCL2 (152 edges), IL10 (152 edges), APOE (149 edges), IL1B (147 edges); (3) SMY-CHD targets: ALB (293 edges), MMP9 (167 edges). The topological property of this network was assessed by network analyzer tool, and the result demonstrates that SMY–CHD PPI network meets the power-law distribution (R^2^ = 0.665, y = 56.438x^−0.739^) (Figure 8), indicating that this is scale-free and has the general characteristic of biological network.

#### Clusters of SMY–CHD PPI network

**Figure 9:**
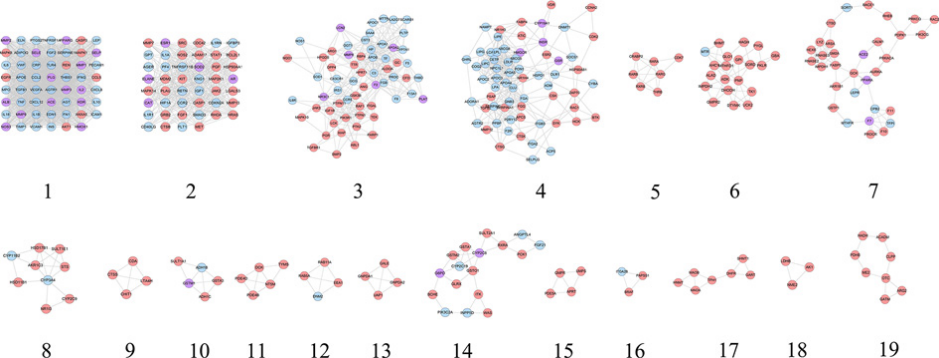
Cluster of SMY–CHD PPI network (pink circle stands for SMY targets; blue circle stands for CHD targets; purple target stands for SMY–CHD targets)

SMY–CHD PPI network was analyzed by cytoscape’s plug-in MCODE and 19 clusters were generated (Table 3 and Figure 9). The targets and genes in top ten clusters were put into DAVID for GO enrichment analysis as an example.

**Table 3:** Clusters of SMY–CHD PPI network

Cluster	Score	Nodes	Edges	Targets and genes
1	48.148	55	1300	CCL2, TGFB1, VCAM1, FGF2, CXCL8, MMP1, IL2, APOE, EGFR, HMOX1, CRP, IFNG, MAPK1, NOS3, CASP3, ADIPOQ, PTGS2, ACE, SERPINE1, SELP, INS, IL6, AGTR1, EDN1, TLR4, VWF, SELE, TNF, ALB, CCL5, MAPK8, MPO, MMP3, TNFRSF1A, PLG, ELN, FN1, VEGFA, IL1B, AKT1, THBS1, PPARG, LEP, PECAM1, AGT, ICAM1, KDR, MMP9, TIMP1, IL10, IL18, ANXA5, MMP2, REN, CXCL12
2	14.489	46	326	CCR2, IGF1, PLAU, MET, FGF1, CTSB, IL1RN, CAT, SMAD3, PF4, KNG1, AR, HIF1A, IL1R1, PGF, GPT, HRAS, MAPK14, TNFRSF11B, GRB2, JAK2, SOD2, MAP2K1, MMP13, ADAM17, IL1A, MMP7, RHOA, NOS2, ESR1, ELANE, SRC, STAT1, HSP90AA1, CD40LG, CDC42, MDM2, BCL2L1, IGFBP3, CASP1, KIT, CDKN2A, AGER, FLT1, LGALS3, RETN
3	9.836	62	300	LCN2, TEK, HP, MTTP, ARG1, TF, FGB, XIAP, MAPK10, GGT1, APOA1, GCG, APOB, ABL1, PGR, APOA2, C3, TGFBR1, PIK3R1, CCR5, CX3CR1, HPGDS, SAA4, DPP4, GSK3B, PLAT, RBP4, PLA2G7, PTPN1, PARP1, TTR, RAF1, PTK2, IRS1, ANG, GC, HPRT1, F13A1, PLTP, PTPN11, JAK3, SOD1, ITGAL, F2, NQO1, F8, ALDOA, LCK, BMP2, THBD, MMP8, APOC1, SCARB1, CYP7A1, CFD, NOS1, F5, IL6R, CST3, NR3C1, PROC, IGF1R
4	9.719	65	311	APCS, AGTR2, SERPINC1, NAMPT, CTSG, NPC1L1, CDK2, NR1H4, LPL, LDLR, APOC3, ITGA2, VDR, RAC1, PON1, COG2, FGG, CSK, CETP, LPA, P2RY12, HMGCR, LIPC, LIPE, APOA5, CCNA2, BTK, INSR, LIPG, ACP5, CTSK, DNMT1, LCAT, CYBA, APOA4, HCK, ATIC, OLR1, FGA, PPBP, SOCS1, CLU, ADM, GSR, APOC2, ABCG5, NR1H3, ADORA1, GNB3, F2R, TGFB2, HSP90AB1, PSAP, MMP12, CYP19A1, ZAP70, SELPLG, SERPINA1, ABCG8, SYK, ITGB3, HSPD1, FABP4, ESR2, GHRL
5	5.333	7	16	RXRB, THRB, RARA, CDK7, CRABP2, RARG, RARB
6	4.526	20	43	GMPR2, ALAD, MTHFD1, ADK, PNP, MTR, BHMT, TK1, DTYMK, GPI, AHCY, PYGL, IMPDH2, PKLR, DHODH, SORD, UCK2, GBA, GLO1, HAGH
7	4.452	32	69	IMPDH1, LYZ, SORT1, PIK3CG, FABP5, AURKA, PRKCQ, RNASE3, CALM1, TFPI, ARSA, BACE1, AKR1B1, F10, RAC2, PPARA, GSTP1, PRKACA, ACE2, PROCR, LEPR, RHEB, MTHFR, PDPK1, GCK, CTSD, CPB2, F7, GM2A, RNASE2, HEXB, F11
8	4.25	9	17	CYP2C9, AKR1C3, CYP3A4, SULT1E1, HSD17B1, NR1I3, CYP11B2, STS, HSD11B1
9	4	4	6	CDA, CTSS, CHIT1, LTA4H
10	4	5	8	GSTA3, SULT1A1, ADH1C, GSTM1, ADH1B
11	4	5	8	PDE4B, DCK, PDE4D, TYMS, NT5M
12	4	4	6	RAB11A, EEA1, RAB5A, DNM2
13	4	4	6	UAP1, GALE, GNPDA1, GNPDA2
14	3.5	17	28	PIK3C2A, GSTM2, PCK1, GLRX, ITK, CYP2C8, BCHE, GSTA1, CYP2C19, G6PD, RXRA, GSTO1, FGF21, INPP5D, SULT2A1, WAS, ANGPTL4
15	3.333	4	5	APRT, GMPR, PDE5A, UMPS
16	3	3	3	ITGA2B, PAPSS1, BRAF
17	3	7	9	DHFR, TPH1, MAOB, GART, HNMT, SHMT1, MAOA
18	3	3	3	LDHB, NME2, AK1
19	2.571	8	9	CLPP, ACADM, GATM, HADH, PDHB, OTC, ME2, ARG2

After GO enrichment analysis, several biological processes were returned. Cluster 1 is mainly related to angiogenesis, smooth muscle proliferation, inflammatory response, blood pressure formation, hypoxia, coagulation, and vascular endothelial cells. Cluster 2 is mainly related to hypoxia, endothelial cell apoptosis, and cell proliferation. Cluster 3 is involved in blood coagulation and lipid metabolism. Cluster 4 is involved in lipid metabolism, inflammation, and coagulation. Cluster 7 is associated with coagulation. Cluster 8 is involved in steroid metabolism and redox. Cluster 10 is associated with redox. Cluster 5, 6 and 9 did not return any CHD-related biological processes. The details were described in Supplementary Table S6.

#### Pathway of SMY–CHD PPI network

**Figure 10:**
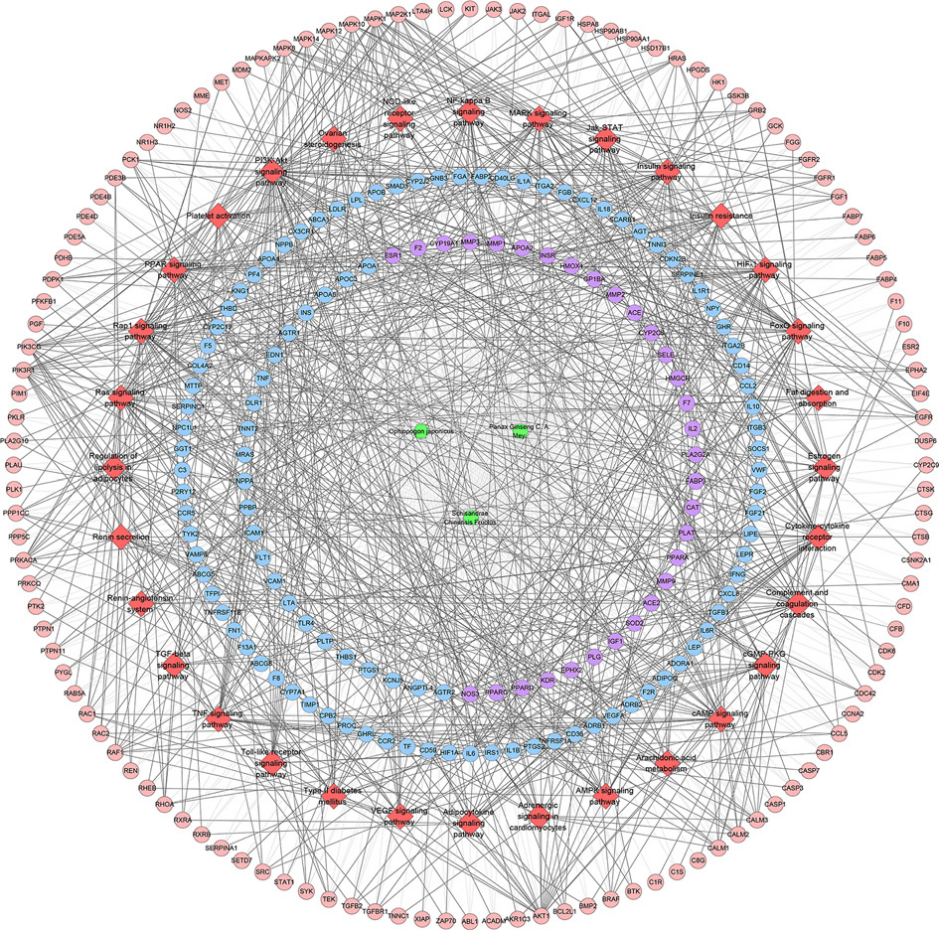
Signaling pathways of SMY−CHD PPI network (red diamond stands for signaling pathway; pink circle stands for SMY targets; blue circle stands for CHD targets; purple target stands for SMY–CHD targets; green hexagon stands for herb. Dark lines stand for relationship among pathways and targets; light lines stand for relationship among herbs and targets)

All interconnected SMY targets, CHD genes and SMY–CHD targets in the network are imported into DAVID for pathway enrichment analysis. After that, 32 signaling pathways were returned. These signaling pathways are arranged according to the degree of enrichment (negative correlation with *P*-value) and count from large to small. The top ten signaling pathways are: Complement and coagulation cascades (*P*=1.92*10^−16^, Count = 30), PPAR signaling pathway (*P*=7.83*10^−14^, Count = 27), HIF-1 signaling pathway (*P*=2.81*10^-11^, Count = 29), FoxO signaling pathway (*P*=7.35*10^−11^, Count = 34), PI3K-Akt signaling pathway (*P*=1.25*10^−10^, Count = 59), Ras signaling pathway (*P*=2.19*10^−10^, Count = 45), Rap1 signaling pathway (*P*=8.56*10^−10^, Count = 42), Insulin signaling pathway (*P*=3.23*10^−09^, Count = 32), VEGF signaling pathway (*P*=8.54*10^−08^, Count = 19), TNF signaling pathway (*P*=2.07*10^−07^, Count = 25), NF-κ B signaling pathway (*P*=3.25*10^−07^, Count = 22) (Figure 10). The details were described in Supplementary Table S7.

### SMY known target–CHD PPI network analysis

#### SMY known target–CHD PPI network

**Figure 11:**
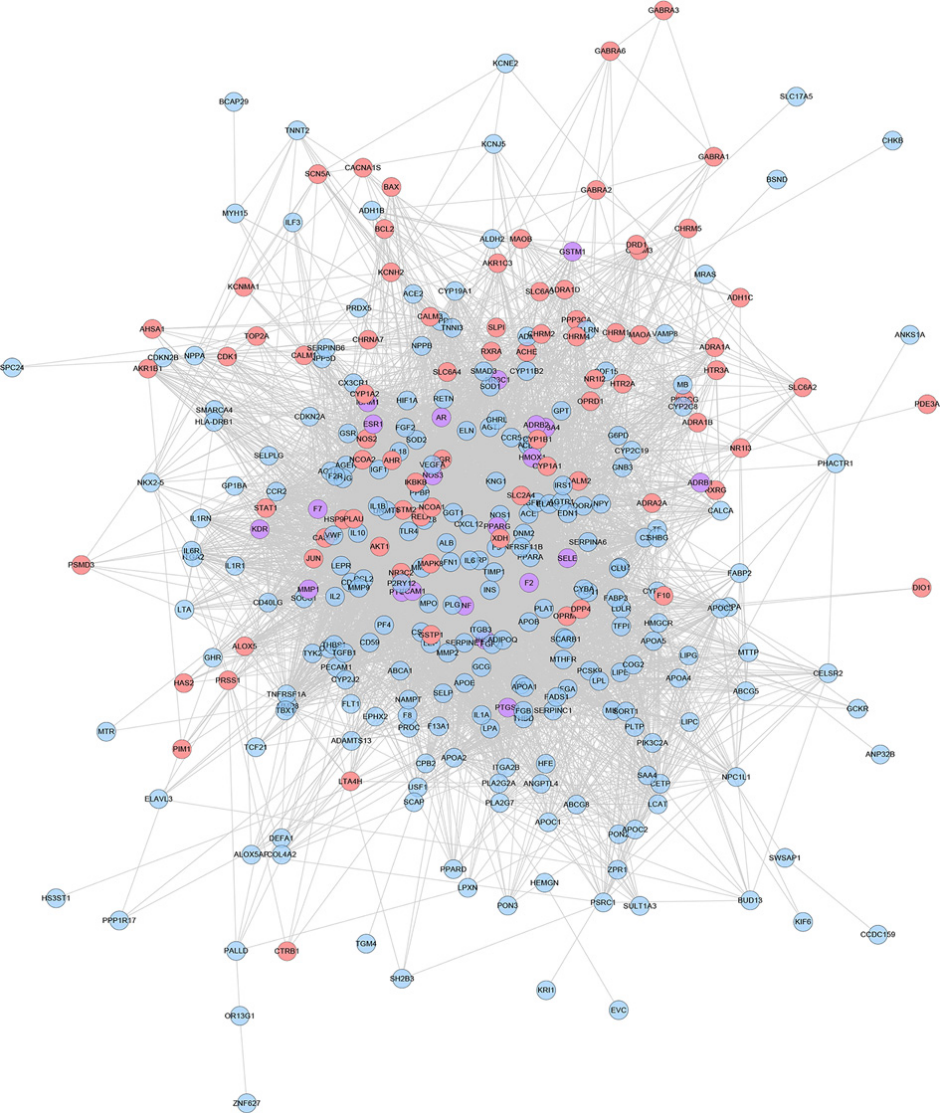
SMY known target–CHD PPI network (pink circle stands for SMY known targets; blue circle stands for CHD targets; purple target stands for SMY known-CHD targets)

**Figure 12:**
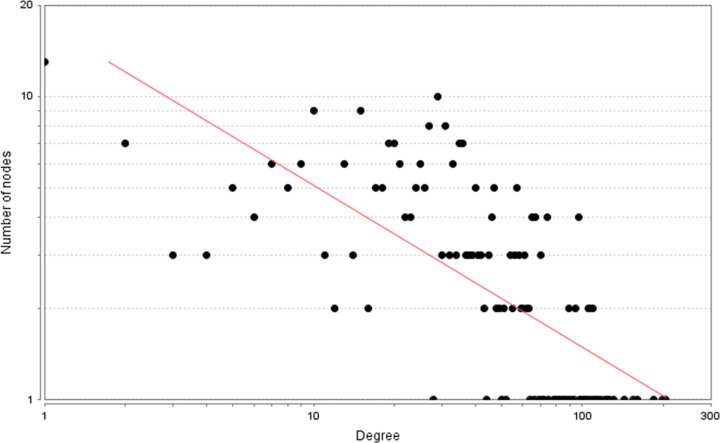
Node degree distribution of SMY–CHD PPI network

The known targets and CHD genes were import into the String database to collect PPI data so as to build the SMY known target–CHD PPI network. This network contains 80 SMY target nodes, 230 CHD gene nodes, 21 SMY known-CHD target nodes, 6683 edges (Figure 11). This network is smaller than the SMY–CHD PPI network and is its authentication network. In this network, some targets appear in the SMY–CHD PPI network can be found. The topological property of this network was assessed by network analyzer tool, and the result demonstrates that SMY knwon-CHD PPI network meets the power-law distribution (R^2^ = 0.502, y = 17.405x^−0.534^) (Figure 12), indicating that this is scale-free and has the general characteristic of biological network.

#### Cluster of SMY known target–CHD PPI network

**Figure 13:**
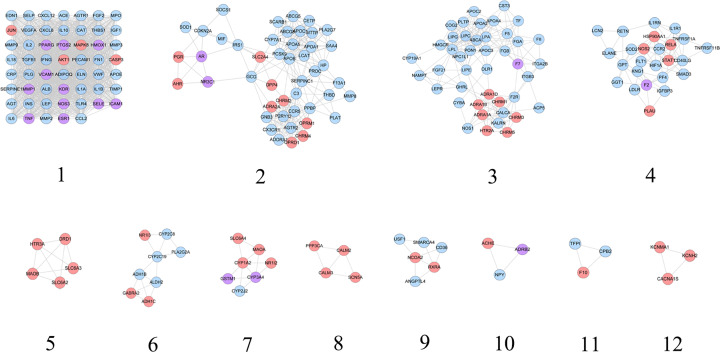
Cluster of SMY–CHD PPI network (pink circle stands for SMY targets; blue circle stands for CHD targets; purple target stands for SMY–CHD targets)

SMY known–CHD PPI network was analyzed by cytoscape’s plug-in MCODE and 12 clusters were generated (Table 4 and Figure 13). The targets and genes in top 5 clusters were put into DAVID for GO enrichment analysis as an example.

**Table 4:** Clusters of SMY known–CHD PPI network

Cluster	Score	Nodes	Edges	Targets and genes
1	47.66	54	1263	TLR4, VWF, SELE, AKT1, TNF, ALB, JUN, CASP3, MAPK8, MPO, MMP3, PLG, FN1, ELN, VEGFA, IL1B, THBS1, PPARG, LEP, PECAM1, AGT, ICAM1, KDR, ESR1, TIMP1, MMP9, IL10, IL18, MMP2, CXCL12, CCL2, IGF1, TGFB1, CAT, VCAM1, FGF2, CXCL8, MMP1, IL2, APOE, HMOX1, CRP, IFNG, NOS3, ADIPOQ, PTGS2, ACE, IL1A, SERPINE1, SELP, INS, IL6, AGTR1, EDN1
2	10	46	225	CDKN2A, PLA2G7, CHRM2, LCAT, NR3C1, PROC, HP, IRS1, MTTP, AGTR2, SERPINC1, PPBP, SOCS1, AHR, AR, SLC2A4, PGR, ABCG5, PCSK9, ADORA1, F13A1, APOA1, GCG, APOB, GNB3, ADRA2A, DPP4, MIF, SOD1, ABCG8, CETP, C3, P2RY12, MMP8, CHRM4, THBD, OPRD1, CCR5, CX3CR1, APOC1, CYP7A1, APOA5, SCARB1, OPRM1, SAA4, PLAT
3	9.571	43	201	ACP5, F5, FGF21, CYBA, CST3, GHRL, APOA4, OLR1, NAMPT, FGA, TF, FGB, NPC1L1, APOC2, CHRM3, CHRM1, LPL, KALRN, PLTP, APOC3, F2R, CALCA, PON1, ABCA1, COG2, ITGA2B, ADRA1A, APOA2, CYP19A1, F8, CHRM5, LPA, LEPR, HMGCR, LIPC, LIPE, HTR2A, ADRA1B, ADRA1D, ITGB3, F7, NOS1, LIPG
4	5.917	25	71	NOS2, FLT1, HSP90AA1, RETN, RELA, CCR2, LCN2, STAT1, IL1RN, SMAD3, PF4, KNG1, HIF1A, TNFRSF1A, IL1R1, GPT, TNFRSF11B, GGT1, LDLR, PLAU, SOD2, F2, ELANE, CD40LG, IGFBP3
5	4.5	5	9	MAOB, SLC6A2, HTR3A, SLC6A3, DRD1
6	3.714	8	13	PLA2G2A, GABRA2, ADH1C, ALDH2, CYP2C8, ADH1B, NR1I3, CYP2C19
7	3.667	7	11	CYP3A4, GSTM1, CYP1A2, NR1I2, SLC6A4, MAOA, CYP2J2
8	3.333	4	5	CALM2, SCN5A, CALM3, PPP3CA
9	3.2	6	8	NCOA2, USF1, ANGPTL4, RXRA, SMARCA4, CD36
10	3	3	3	ADRB2, NPY, ACHE
11	3	3	3	CPB2, F10, TFPI
12	3	3	3	CACNA1S, KCNMA1, KCNH2

After GO enrichment analysis, several biological processes were returned. Cluster 5 did not return CHD-related biological processes. Take cluster 1 as the example:

The genes in Cluster 1 mainly focus on GO:0045429, GO:0048661, GO:0002576, GO:0006954, GO:0001525, GO:0001666, GO:0043066, GO:0031663, GO:0071222, GO:0045766, GO:0010628, GO:0030198, GO:0008217, GO:0014068, GO:0008284, GO:0070374, GO:0022617, GO:0006955, GO:0051092, GO:0050901, GO:0001938, GO:0071260, GO:0050729, GO:0010595, GO:0043536, GO:0051781, GO:0008285, GO:0071356, GO:0032757, GO:0045840, GO:0007155, GO:0010888, GO:0051384, GO:0050900, GO:0034374, GO:0032355, GO:0043537, GO:0032755, GO:0071456, GO:0032729, GO:0051044, GO:1904707, GO:0044130, GO:0042593, GO:0000165, GO:0000187. The details were described in Supplementary Table S8.

#### Pathways of SMY known target–CHD PPI network

**Figure 14:**
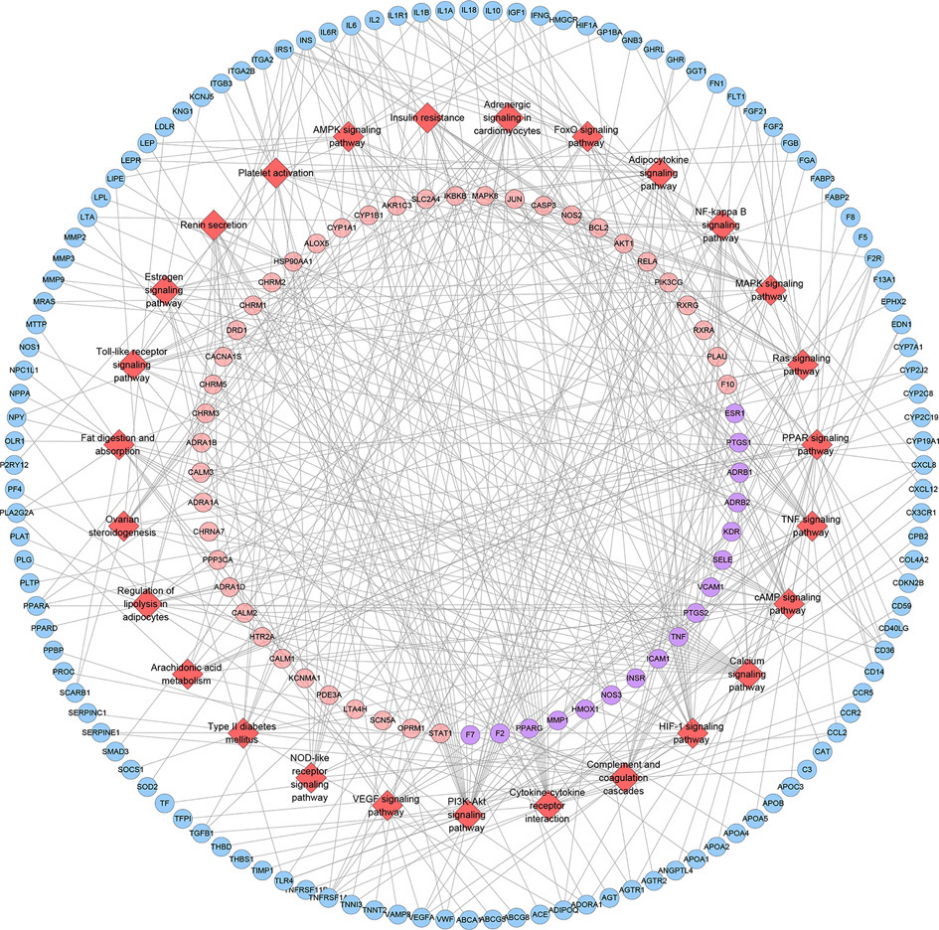
Signaling pathways of SMY known-CHD PPI network (red diamond stands for signaling pathway; pink circle stands for SMY known targets; blue circle stands for CHD targets; purple target stands for SMY known-CHD targets)

All interconnected SMY known targets, CHD genes and SMY known-CHD targets in the network are imported into DAVID for pathway enrichment analysis. After that, 27 signaling pathways were returned. These signaling pathways are arranged according to the degree of enrichment (negative correlation with *P*-value) and count from large to small. The top 10 signaling pathways are: (hsa04610) Complement and coagulation cascades, (hsa03320) PPAR signaling pathway, (hsa04066) HIF-1 signaling pathway, (hsa04668) TNF signaling pathway, (hsa04920) Adipocytokine signaling pathway, (hsa04064) NF-κ B signaling pathway, (hsa04975) Fat digestion and absorption, (hsa04913) Ovarian steroidogenesis, (hsa04151) PI3K-Akt signaling pathway, (hsa04020) Calcium signaling pathway (Figure 14). The details were shown in Supplementary Table S9.

### Effects of SMI on the myocardial enzymes in rats

**Figure 15:**
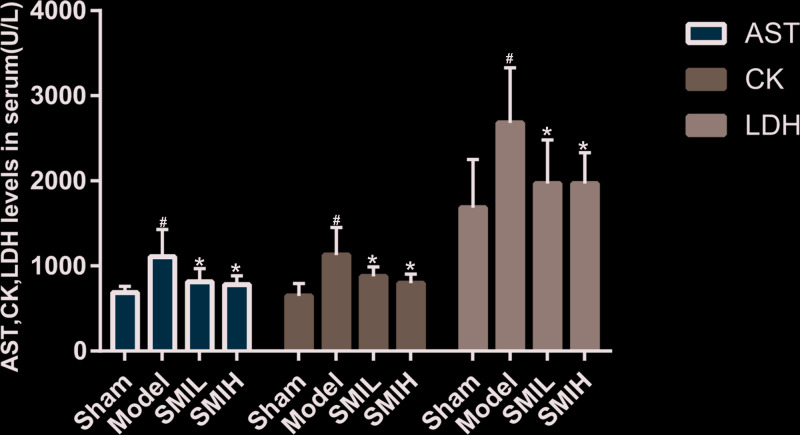
Effects of SMI on the myocardial enzymes in rats (compared with sham operation group ^#^*P*<0.01; compared with model group *P<0.05)

Compared with the sham operation group, the activities of serum AST, CK and LDH in the model group were significantly increased (*P*<0.01). Compared with the model group, the SMI (low dose and high dose) can reduce the activities of serum AST, CK and LDH in rats (*P*<0.05). The difference of serum AST, CK and LDH between SMIL group and SMIH group was of no statistical significance (*P*>0.05) (Figure 15).

### Histopathological changes of myocardial tissue in rats

**Figure 16:**
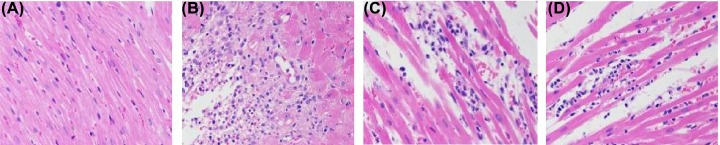
The Effects of SMI on myocardial tissue Histopathological changes of myocardial tissue in rats (HE staining, 400×) ((**A**): sham operation group; (**B**) model group; (**C**) SMIL group; (**D**) SMIH group).

In the sham operation group, the myocardial fibers were uniformly colored, arranged neatly and regularly, with clear cell boundaries and no abnormalities in the stroma. In the model group, myocardial fibers showed a large number of coagulative necrosis accompanied by severe neutrophil punctate infiltration, myocardial fiber edema, and sarcoplasma is looseness and light staining. The degree of myocardial fiber necrosis and neutrophil spot infiltration in the SMI low and high dose groups were significantly reduced compared with the model group, and only part of the myocardial fiber was edema. There is no obvious difference between SMIL group and SMIH group (Figure 16).

### Myocardial injury score

**Figure 17:**
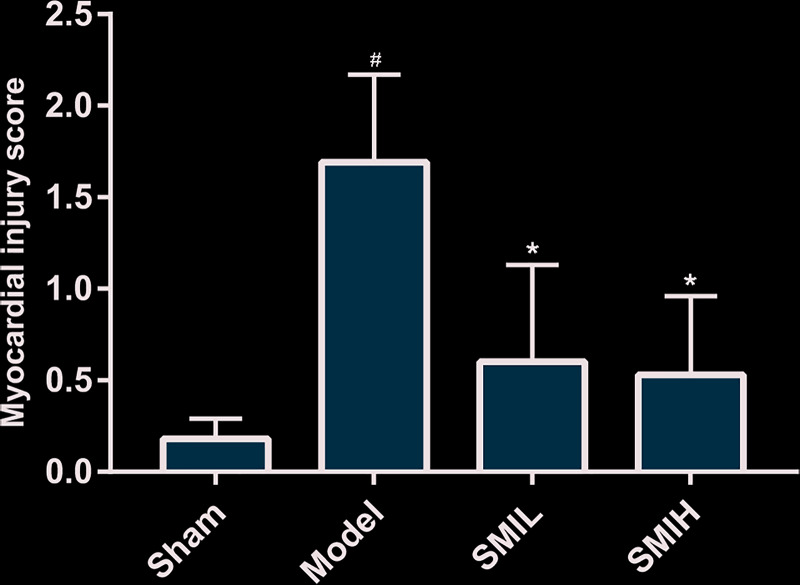
Myocardial injury score in rats (compared with sham operation group ^#^*P*<0.05; compared with model group *P<0.05)

Compared with the sham operation group, the myocardial morphology scores in the model group were significantly increased (*P*<0.01); while the difference between the low and high SMI groups and the sham operation group was not statistically significant (*P*>0.05). Compared with the model group, the scores of the SMI low and high dose groups were significantly reduced (*P*<0.01). The difference of myocardial injury score between SMIL group and SMIH group was of no statistical significance (*P*>0.05) (Figure 17).

### Effects of SMI on serum LPO, SOD and GSH-Px in rats

**Figure 18:**
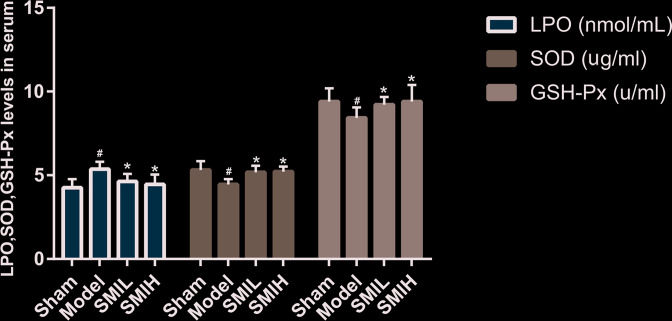
Effects of SMI on serum LPO, SOD and GSH-Px in rats (compared with sham operation group ^#^*P*<0.05; compared with model group *P<0.05)

Compared with the sham operation group, the serum LPO content was significantly increased, and SOD and GSH-Px activities were significantly reduced in the model group (*P*<0.01). Compared with the model group, SMI (low and high dose) can reduce serum LPO content and increase serum SOD and GSH-Px activities in myocardial infarction rats (*P*<0.05). The difference of serum LPO, SOD and GSH-Px between SMIL group and SMIH group was of no statistical significance (*P*>0.05) (Figure 18).

### Expression of MMP-9 mRNA and TIMP-1 mRNA in myocardial tissue

**Figure 19:**
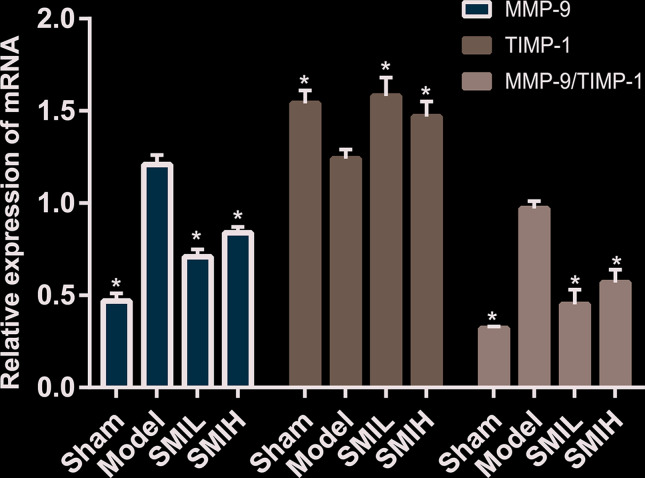
Expression of MMP-9 mRNA and TIMP-1 mRNA in myocardial tissue (compared with model group *P<0.01)

The results showed that MMP-9 mRNA expression in the sham operation group was the least (*P*<0.01). The expression of MMP-9 mRNA and the ratio of MMP-9 mRNA/TIMP-1 mRNA in the model group were significantly higher than those in the sham operation group (*P*<0.01). Compared with the model group, the expression of MMP-9 mRNA and the ratio of MMP-9 mRNA/TIMP-1 mRNA in SMIL group and SMIH group were lower (*P*<0.01). Compared with the model group, the expression of TIMP-1 mRNA was significantly increased in SMIL group and SMIH group (*P*<0.01). The expressions of MMP-9 mRNA and TIMP-1 mRNA and the ratio of MMP-9 mRNA/TIMP-1 mRNA in the SMIL group were similar to those in SMIH group (*P*>0.05) (Figure 19).

## Discussion

In this research, three major networks (CHD genes’ PPI network, SMY–CHD PPI network, SMY known target–CHD PPI network) and several minor networks (generated by analyzing the three major networks, such as pathway of CHD genes’ PPI network) were constructed. The first network revealed a possible mechanism for CHD. The second network explores possible mechanisms for SMY in treating CHD. The third network is a verification of the second network.

The current pathogenesis of CHD mainly includes inflammation, hemorheology, endothelial damage, thrombosis, smooth muscle cloning and so on [46,46–47]. Studies on inflammation suggest that if various risk factors for atherosclerosis persist, it will cause damage to endothelial cells [48], and then inflammatory reactions will occur. During this process, a variety of inflammatory factors (including interleukin-6 [IL-6], CRP, complement, CD40 and myeloperoxidase [MPO]) promoted the development of AS, and gradually lead to fatty streaks, plaques, and atheromatous plaques, eventually causing CHD [49–51]. This research shows that SMY can inhibit the role of inflammation-related modules (namely, clusters) in the complex network of CHD.

The theory of blood flow pathology believes that CHD is related to increased blood viscosity, because too high blood viscosity can increase the resistance of the heart microcirculation, which will cause ischemia and hypoxia in the corresponding blood supply part, promote the formation of thrombus, and result in the occurrence of CHD [52]. Blood viscosity is inversely related to cardiac blood flow [52]. If patients with CHD are complicated by hyperlipidemia, the blood vessel endothelium will be damaged due to the lipid adhesion and the intima of the coronary arteries [53]. This will result in abnormal coronary hemorheology, and the blood viscosity will increase accordingly, which will eventually reduce the myocardial blood and oxygen supply [53]. If the risk factors persist, the above processes will continue to interact, forming a vicious cycle, which will make the ischemic myocardial disease more and more serious [54]. This research shows that SMY can regulate coagulation-related modules of CHD and biological processes such as platelet activation and degranulation (such as GO:0002576, GO:0030168, hsa04611).

The endothelial injury hypothesis believes that endothelial injury will reduce the production of nitric oxide (NO) and produce many adhesion molecules, such as intercellular adhesion molecule (ICAM-1) and vascular cell adhesion molecule-1 (VCAM-1). This will accelerate the adhesion of monocytes to endothelial cells and smooth muscle cells, and promote their migration and proliferation, eventually leading to intimal thickening and atherosclerosis [55,56]. Patients with hyperlipidemia often experience intravascular lipid deposition, which is also one of the causes of endothelial damage, and the consequence is the generation of atheromatous plaque in the blood vessels. Lipid deposition also increases the permeability of the vascular endothelium, which further exacerbates the endothelium damage. With the deposition of lipid and the formation of oxidized low-density lipoprotein (oxLDL-c), leukocytes and monocytes in the blood are transformed into macrophages. Macrophages, foam cells, and the substances released during their apoptosis form the lipid core [57,58]. T cells are activated and collagen fibers proliferate, covering the surface of the lipid core, forming a fibrous cap [59]. In addition, the vascular smooth muscle cell (VSMC) in the vascular media is stimulated by various risk factors, which causes the proliferation and migration of SMC [60,61]. As the number of SMCs continues to increase, a large amount of lipids are wrapped by fibrous connective tissue secreted by SMCs, thereby worsening atherosclerosis [60,61]. Meanwhile, this research shows that SMY can regulate endothelial cells and their adhesion factors (such as GO:0007155, GO:0001938, hsa04060), and can regulate foam cell formation-related (such as GO:0010745, GO:0010744), lipid metabolism-related (such as cluster 4), and smooth muscle cell-related (such as GO:0048661, GO:1904707) modules. Recent studies have shown that SMI can improve endothelial damage in patients with CHD [62] and can reduce liver lipids and lipid peroxidation in rats fed a high cholesterol diet [63].

Oxidative stress is also involved in the pathological process of CHD. When endothelial cells are damaged and cause an inflammatory response, on the one hand, they attract neutrophils and monocytes to the injured area to release inflammatory mediators; on the other hand, they induce macrophages, vascular endothelial cells to produce and release NO, and neutrophils to produce a large number of oxygen free radicals, leading to infiltration of leukocytes, deposition of inflammatory products and accumulation of oxygen free radicals, participating in atherosclerotic plaque formation [64–66]. This research shows that SMY regulates oxidative stress modules in a complex network of CHD, such as GO:0001666, GO:0006979.

Our experimental studies also show that SMI can significantly reduce the area of myocardial infarction and reduce serum AST, CK and LDH activities, suggesting that it has a significant protective effect on ischemic myocardium. SMI can also reduce serum LPO content and increase SOD and GSH-Px activity; among them, LPO is the product of free radical-induced lipid peroxidation. Oxygen free radicals produced by ischemic myocardium can enter the blood circulation and increase the content of oxygen free radicals in peripheral blood. Therefore, the degree of damage caused by oxygen free radicals to the body can be assessed indirectly through changes in LPO content and changes in endogenous antioxidant enzymes (SOD, GSH-Px) activity. Because SMI has a regulating effect on these contents, it suggests that SMI may reduce the damage of myocardial oxygen by inhibiting the lipid peroxidation process through a certain mechanism, and at the same time, increasing the activity of endogenous antioxidant enzymes. Such antioxidant effect may also be one of its mechanisms for protecting ischemic myocardium. Meanwhile, studies showed that SMY can inhibit calcium overload and the increase in oxygen free radicals in myocardial cells after myocardial infarction, the mechanism of which may be that SMY can up-regulate the expression of SERCA2 and RyR2 mRNA in myocardial cells [67]. Guo et al. found that the protective effects of SMY in myocardial ischemic injury rats are as follows: (1) SMY inhibited the biological process of oxidative stress by regulating the levels of CAT, GSH-Px, MDA and NOX in serum; (2) SMI played an antioxidant role through the Nrf2 pathway and its downstream genes (Nrf2, HO-1, CAT, SOD1, GPX1, NQO1, et al.); (3) both *in vivo* and *in vitro* experiments showed that SMY can regulate the energy metabolism and mitochondrial respiratory function of myocardial ischemic rats [68]. Zheng et al. found that SMY extract and its monomer components can effectively inhibit cardiomyocyte apoptosis in high glucose environment, and its anti-apoptotic mechanism may be related to blocking mitochondrial apoptosis pathway (Fas/FasL pathway and p53-Bcl2/Bax pathway) [69]. The shortcoming of this study is that the systematic pharmacology and chemoinformatics strategy cannot directly reflect the effect of compound content on organisms, hence, experiments are still needed to verify the prediction results of systematic pharmacology and chemoinformatics. However, this does not mean that this research strategy is useless. Future research can carry out experimental pharmacological verification based on the prediction results of systematic pharmacology and chemoinformatics strategy so as to make the experimental results more accurate and reliable.

## Conclusions

SMY may regulate the signaling pathways (such as PPAR, FoxO, VEGF signaling), biological processes (such as angiogenesis, blood pressure formation, inflammatory response) and targets (such as AKT1, EGFR, MAPK1) so as to play a therapeutic role in CHD.

## Supplementary Material

Supplementary Tables S1-S9

## Data Availability

The data that support the findings of the present study are openly available in Supplementary Materials.

## Abbreviations

AST: aspartate aminoconvertase
ADME: absorption, distribution, metabolism and excretion
CHD: coronary heart disease
CRP: C-reactive protein
GO: Gene Ontology
DAVID: The Database for Annotation, Visualization and Integrated Discovery
GSH-Px: glutathione peroxidase
GAPDH: glyceraldehyde-3-phosphate dehydrogenase
HE: Hematoxylin–Eosin
HIF-1: hypoxia inducible factor-1
IL-6: interleukin-6
LDH: lactate dehydrogenase
LPO: lipid peroxide
NO: nitric oxide
PPI: protein–protein interaction
SMI: Shengmai injection
SMIH: SMI high-dose group
SMIL: SMI low-dose group
SMY: Shengmai Yin
SOD: superoxide dismutase
TCM: traditional Chinese medicine
TCMID: traditional Chinese medicine integrative database
TCMSP: Traditional Chinese Medicine Systems Pharmacology Database
VEGF: vascular endothelial growth factor
